# Tibetan sheep grazing modifies rodent density and their interactions effect on GHG emissions of alpine meadow

**DOI:** 10.1038/s41598-019-53480-z

**Published:** 2019-11-19

**Authors:** Yingxin Wang, Hang Yuan, Xinglu Zhang, Yi Sun, Shenghua Chang, Guang Li, Fujiang Hou

**Affiliations:** 1 State Key Laboratory of Grassland Agro-ecosystems, Key Laboratory of Grassland Livestock Industry Innovation, Ministry of Agriculture and Rural Affairs; College of Pastoral Agriculture Science and Technology, Lanzhou University, Lanzhou, 730020 China; 2 College of Forestry, Gansu Agricultural University, Gansu Provincial Key Laboratory of Arid land Crop Science, Lanzhou, 730070 China

**Keywords:** Ecosystem ecology, Environmental impact

## Abstract

Digging and mound-building by rodents lead to considerable disturbances in the topsoil and may affect plant composition, soil properties. However, little is known about the effects of these activities on GHG emissions, especially under different grazing management. This paper aimed to measure changes in CO_2_ and CH_4_ efflux with varying grazing management during the warm and cold seasons and to relate CO_2_ and CH_4_ efflux to pika burrow density and zokor mound density with different grazing management. Results of this study showed that CO_2_ efflux was significantly affected by the grazing season, whereas CH_4_ efflux was significantly affected by the grazing system. There were significant relationships between GHG efflux and rodent population density which were regulated by grazing management. CO_2_ efflux increased linearly with rodent density under seasonal continuous grazing in warm season. CO_2_ and CH_4_ efflux and rodent population density showed a significant quadratic convex relationship under rotational grazing at 24 SM/ha in warm and cold seasons and rotational grazing at 48 SM/ha in cold season. Under rotational grazing at light stocking rate (24 SM/ha), appropriate populations of rodents were beneficial for decreasing GHG emissions. This results also used to help drive a best-practices model for grazing practices of local herders.

## Introduction

Rodents tend to live in large populations and are widely distributed throughout the world; they also play a major role in the structure and function of many ecosystems^1,2^. Rodent ecological disturbances can be classified into at least four primary categories: tunnel digging, foraging, feces deposition, and urine deposition^3–5^. Tunnel digging is the primary rodent activity that affects ecosystems^6^. Digging and mound-building by rodents lead to considerable disturbances in the topsoil and may affect plant composition, soil properties, and GHG emissions^7,8^.

The plateau pika (*Ochotona curzoniae*) and plateau zokor (*Myospalax baileyi*) are key native species unique to the grassland landscape of Qinghai Tibet Plateau (QTP)^3,9^. Their population density is controlled by predation by weasels, polecats, foxes, wolves, and eagles^3^. On the one hand, their digging activity modifies the environment by enhancing the ability of soil to absorb precipitation, contributing to nutrient cycling, and creating microhabitats, all of which in increased plant species richness. On the other hand, they compete with livestock for scarce food resources, and their digging destroys the sod layer and buries vegetation under the excavated soil^10^. An adult pika consumes about 77.3 g of fresh grass per day, which is about 50% of its body weight. The food intake of 56 adult pikas equals that of one Tibetan sheep^11^. It has been estimated that rodents consume 15,000 billion kg of grassland foliage on the QTP each year^12^. Because zokors dig below ground and also build mounds on the surface, their constant activity modifies different soil layers, facilitating the movement of air, water, nutrients and contaminants in solution, and other organisms^13,14^. Areas with a high mound density can provide an ecological opportunity, as they are conducive to colonization by plant species^9^. Zokor mounds directly alter the grassland microtopography and redistribute soil moisture and heat, affecting the pattern of plant distribution.

The QTP is a vast geographic area that accounts for 36.5% of the total grassland and 25.6% of the soil organic carbon (SOC) stock in China^15,16^. It is currently subject to warming^17,18^, and an increase in GHGs, particularly carbon dioxide (CO_2_) and methane (CH_4_), has been identified as the main contributor to this warming^19,20^. Most, if not all, prior field studies of GHG emissions in the QTP grassland were performed using small plots (5 m × 5 m) without grazing livestock, pikas, or zokors^21^. Most grassland ecosystems on the QTP are subjected to varying degrees of biomass consumption by both domestic livestock and indigenous herbivores, most notably pika and zokor, and this may have important effects on ecosystem CO_2_ and CH_4_ efflux. Rodent activity around burrow holes and mounds disturbs soil structure, which makes organic carbon available to microbial decomposition, causing an increase in ecosystem CO_2_ emission^8^. CH_4_ uptake by upland soil is a biological process governed by the availability of CH_4_ and oxygen as well as the activity and quantity of methanotrophic bacteria in the soil^7^. Improvements in soil gas permeability and aeration and a decrease in soil moisture on zokor mounds facilitates the diffusion of atmospheric CH_4_ and oxygen into the soil. Having sufficient CH_4_ and oxygen substrates for methanotrophic bacteria in zokor mounds enhances atmospheric CH_4_ uptake^22^. However, there is currently little data estimating the role of pika and zokor on CO_2_ and CH_4_ efflux in their indigenous areas^8,23^.

In this study, the chamber method was used to measure CO_2_ and CH_4_ efflux in alpine meadow of the QTP with different plateau pika and zokor densities (as determined by burrow and mound numbers) and under different grazing practices during both the warm and cold seasons in 2010 and 2011. To our knowledge, this is the first pastoral study on the QTP to attempt a complete and controlled assessment of GHG emissions taking into account populations of both livestock and native rodents. The objectives of this study were (1) to measure changes in GHG emissions (CO_2_ and CH_4_ efflux) and rodent density (pika burrows and zokor mounds) with varying grazing management during the warm and cold seasons and (2) to relate CO_2_ and CH_4_ efflux to pika burrows and zokor mounds with different grazing management during the warm and cold seasons. The hypotheses were i) GHG emissions and rodent density were both effected by grazing management; ii) relationship between GHG emissions and rodent density were complex and these relationships were regulated by grazing management.

## Results

### Effects of grazing management on vegetation and soil properties

**Figure 1:**
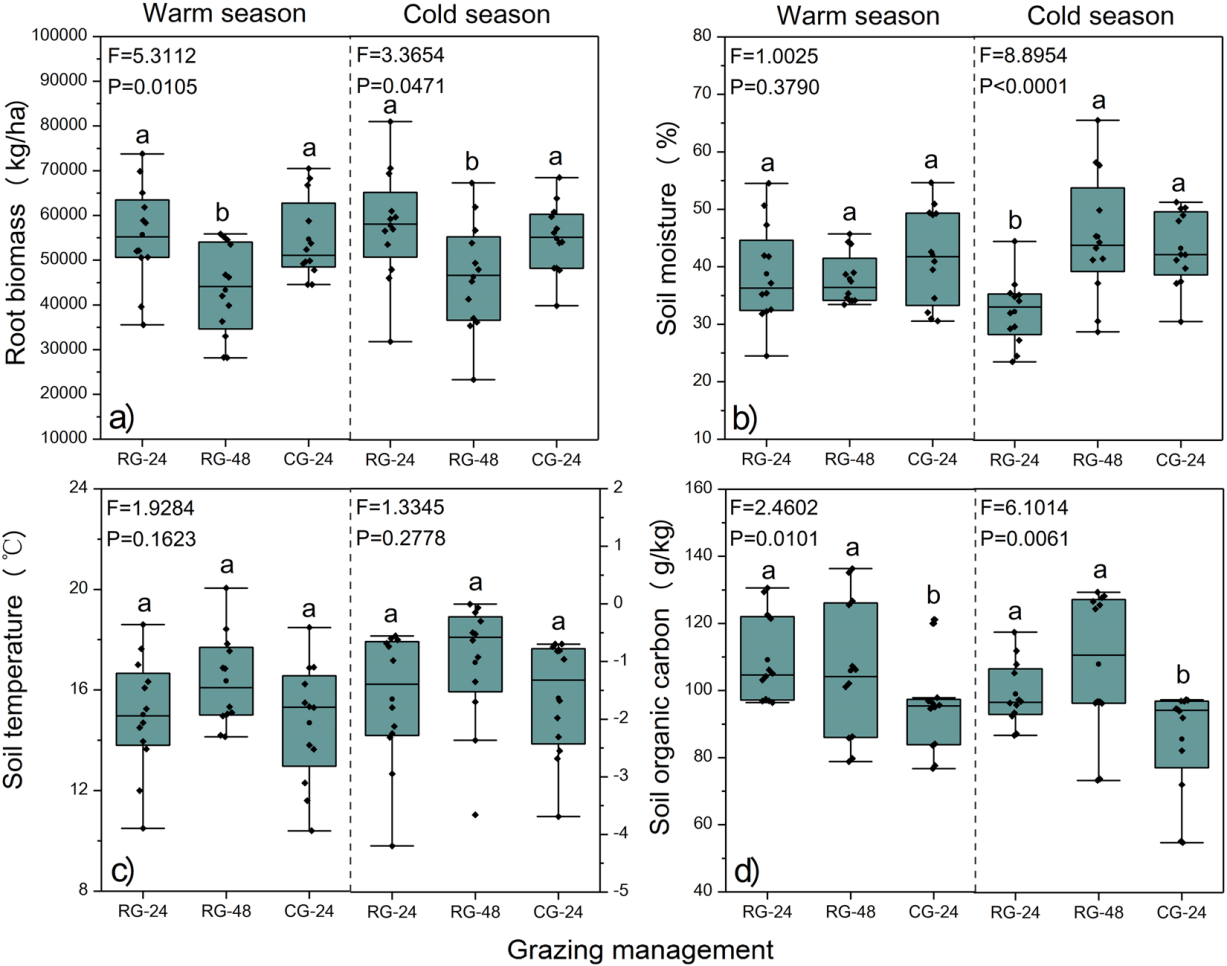
Effects of grazing management on root biomass, soil moisture, soil temperature, and SOC in warm and cold seasons. Alpine meadows were rotationally grazed by Tibetan sheep at stocking rates of 24 SM/ha (RG-24) and 48 SM/ha (RG-48) or were continuously grazed at 24 SM/ha (CG-24). Pairs of dissimilar letters indicate significant differences (P< 0.05) between grazing management.

Root biomass under the RG-48 treatment was significantly (*P* < 0.05) higher than that under RG-24 and CG-24 treatments in both warm and cold seasons (Fig. 1a). Soil moisture under RG-24 was significantly (*P* < 0.05) higher than that under RG-48 and CG-24 treatments in cold season but not in warm season (Fig. 1b). There was no significant (*P* > 0.05) difference for soil temperature between grazing management (Fig. 1c). Soil organic carbon under the CG-24 treatment was significantly (*P* < 0.05) lower than that under RG-24 and RG-48treatments in both warm and cold seasons (Fig. 1d).

### Effects of grazing management on CO_2_ and CH_4_ efflux

**Figure 2:**
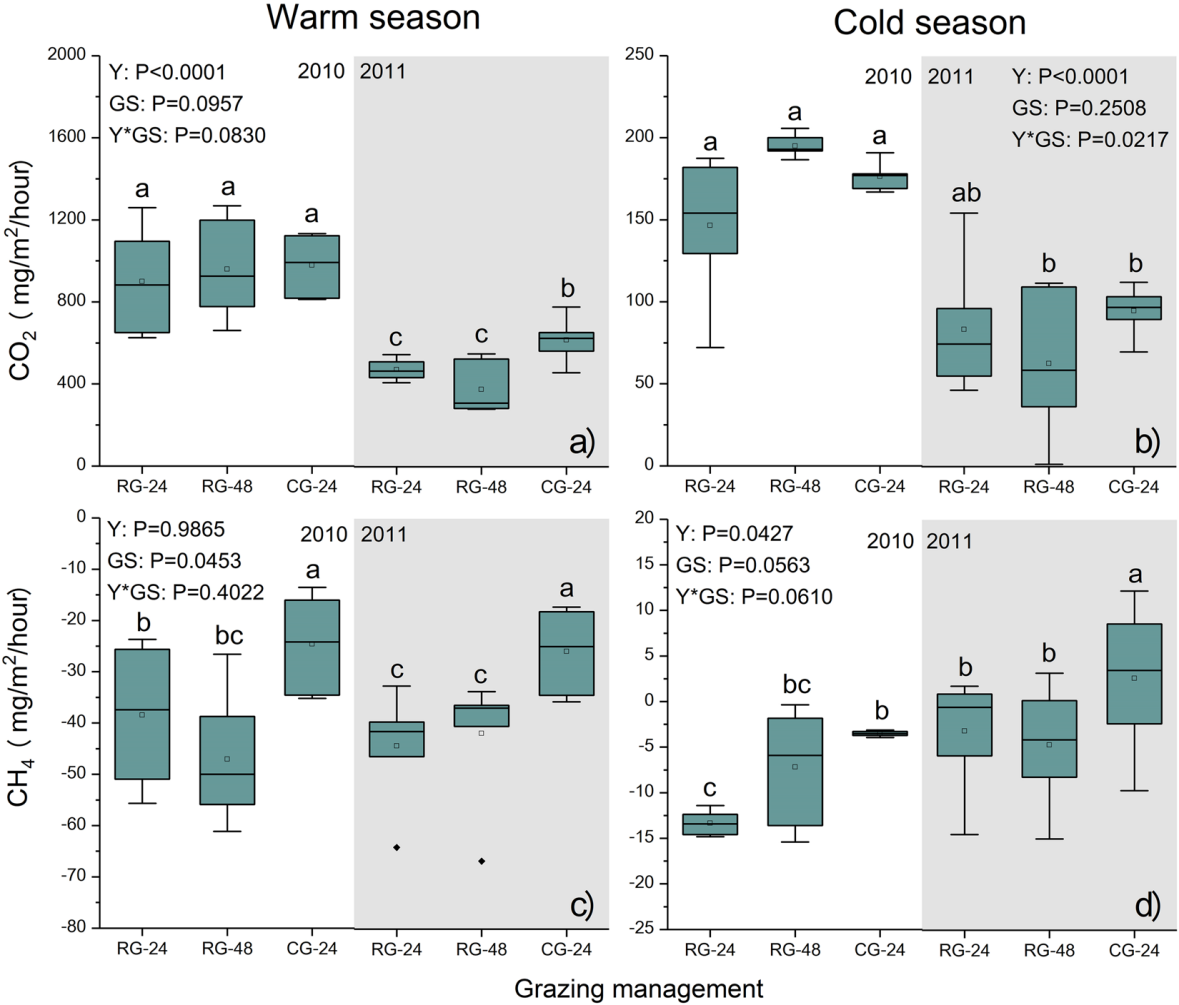
Effects of grazing management on CO_2_ and CH_4_ efflux during the warm and cold seasons in 2010 and 2011. Alpine meadows were rotationally grazed by Tibetan sheep at stocking rates of 24 SM/ha (RG-24) and 48 SM/ha (RG-48) or were continuously grazed at 24 SM/ha (CG-24). Y, year; GS, grazing management; Y*GS, interaction of Y and GS. Pairs of dissimilar letters indicate significant differences (P < 0.05) between grazing management.

Ecosystem CO_2_ efflux was significantly higher in 2010 than in 2011 (*P* < 0.05) for all grazing management during both warm and cold seasons (Fig. 2a,b). There was no significant difference in CO_2_ efflux among grazing management during either season in 2010 (Fig. 2a,b). CO_2_ efflux under CG-24 treatment was significantly (*P* < 0.05) higher than that under RG-48 and RG-24 treatments in warm season in 2011 (Fig. 2a). Ecosystem CH_4_ efflux was significantly higher (*P* < 0.05) with continuous grazing (CG-24) than with either rotational grazing treatment (RG-24 and RG-48) during the warm season in both 2010 and 2011 (Fig. 2c). During the cold season of 2010, CH_4_ efflux under the RG-24 treatment was significantly lower (*P* < 0.05) than that under the CG-24 and RG-48 treatments (Fig. 2d). During the cold season in 2011, CH_4_ efflux under both RG-24 and RG-48 treatments was significantly lower (*P* < 0.05) than that under the CG-24 treatment (Fig. 2d).

### Effects of grazing management on rodent population density

**Figure 3:**
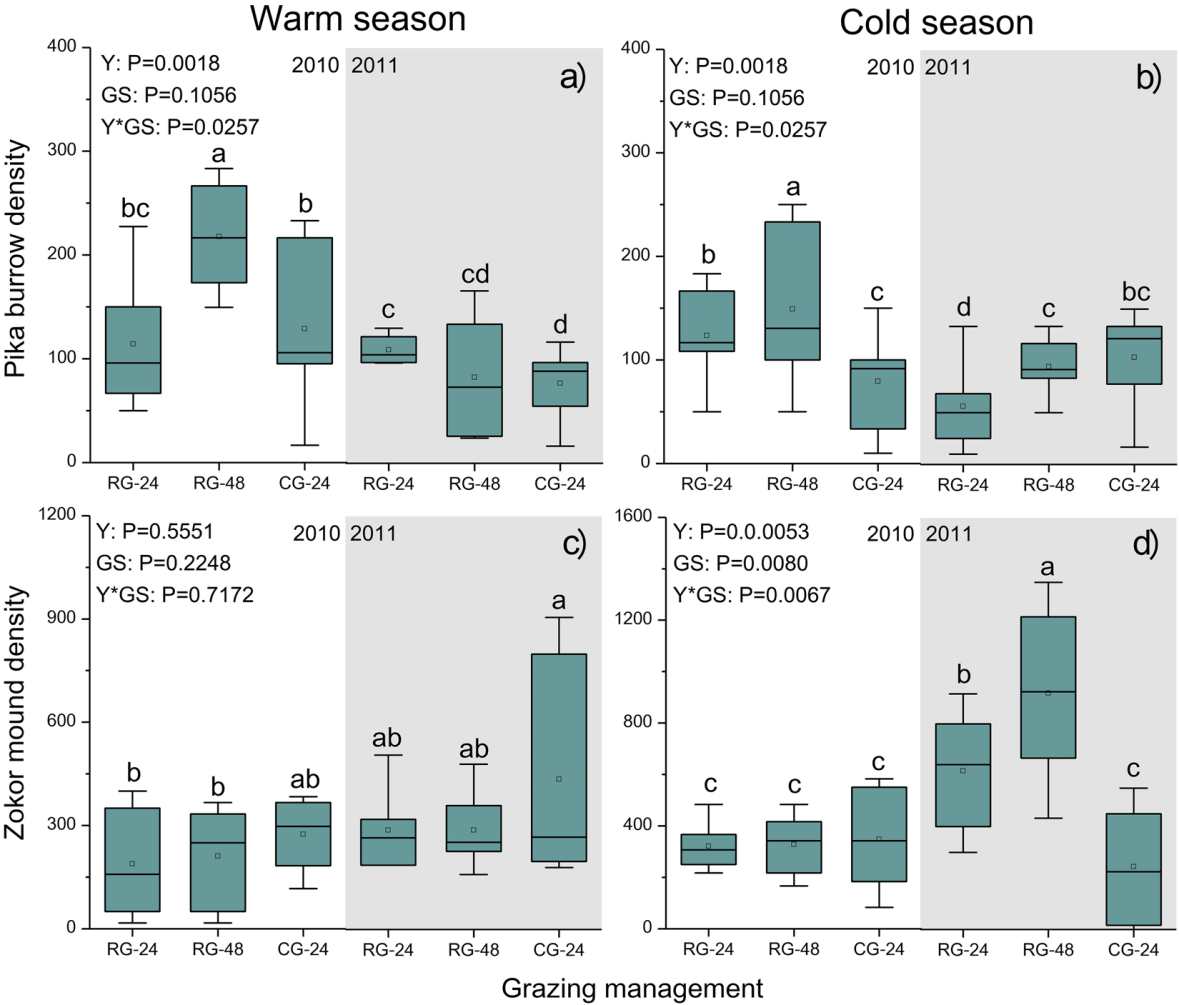
Effects of grazing management on pika burrow and zokor mound density during the warm and cold seasons in 2010 and 2011. Alpine meadows were rotationally grazed by Tibetan sheep at stocking rates of 24 SM/ha (RG-24) and 48 SM/ha (RG-48) or were continuously grazed at 24 SM/ha (CG-24). Y, year; GS, grazing management; Y*GS, interaction of Y and GS. Pairs of dissimilar letters indicate significant differences (P < 0.05) between grazing management.

Pika burrow density for all grazing management and both seasons were significantly higher (*P* < 0.05) in 2010 than in 2011, with the exception of the CG-24 treatment during the cold season (Fig. 3a). During both seasons of 2010, pika burrow density under the RG-48 treatment was significantly higher (*P* < 0.05) than that under the RG-24 and CG-24 treatments (Fig. 3a,b). In 2011, pika burrow density under the RG-24 treatment was significantly higher (*P* < 0.05) than that under the RG-48 and CG-24 treatments during the warm season (Fig. 3a) but was significantly lower (*P* < 0.05) during the cold season (Fig. 3b).

In 2010, there was no significant difference (*P* > 0.05) in zokor mound density among grazing systems during either season (Fig. 3c,d). During the warm season of 2011, zokor mound density under CG-24 treatment was higher than under RG-24 and RG-48 treatments (Fig. 3c). During the cold season of 2011, mound density under the RG-48 treatment was significantly higher (*P* < 0.05) than that under the RG-24 and CG-24 treatments (Fig. 3d).

### Relationship between rodent density and GHG emissions

**Figure 4:**
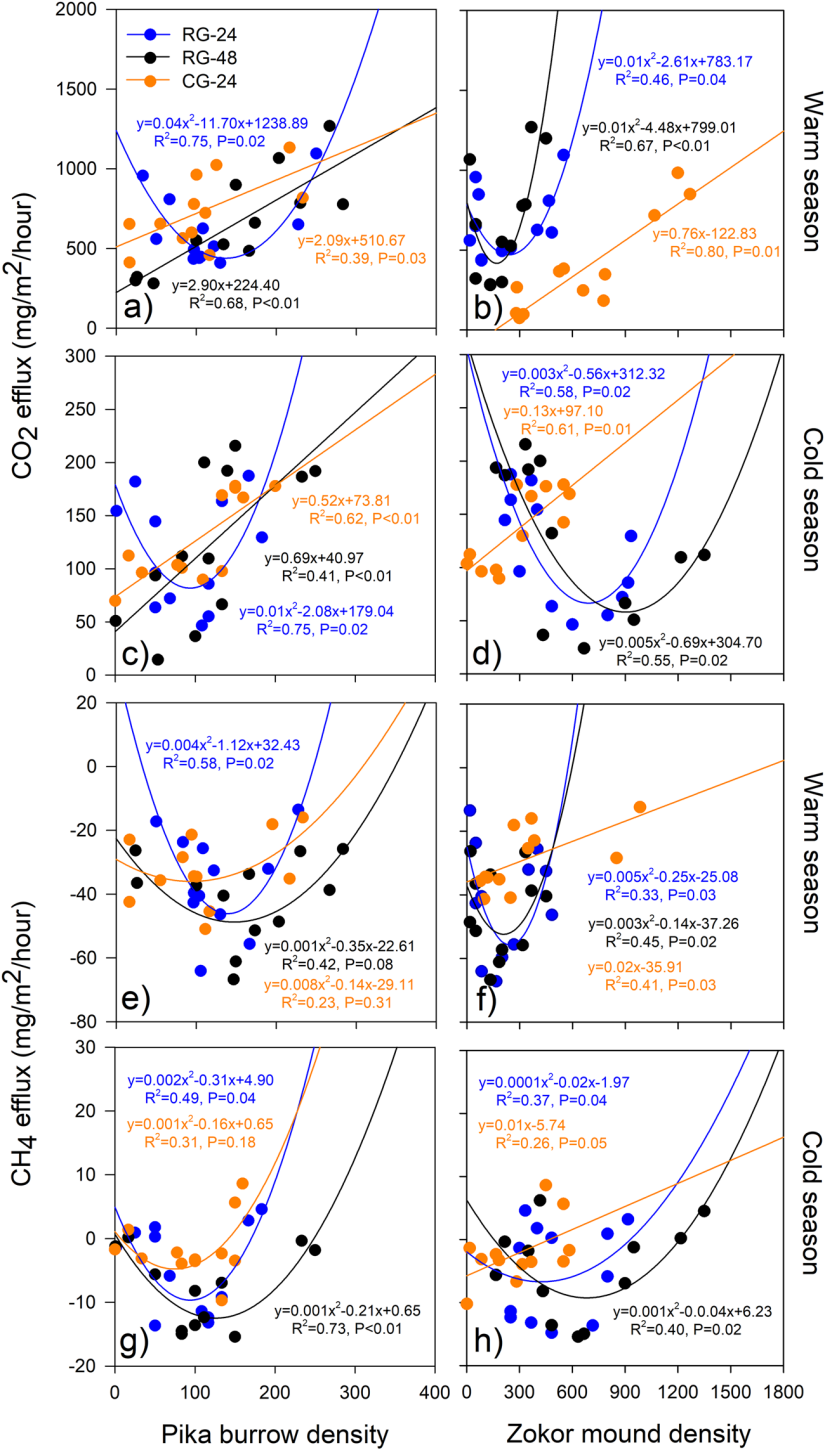
Relationships between CO_2_ and CH_4_ efflux and rodent density (pika burrow and zokor mound density) under different grazing systems during the warm and cold seasons. Alpine meadows were grazed as in Fig. 1. Lines denote the linear fit.

CO_2_ efflux and pika burrow density showed significant positive linear relationships under the RG-48 and CG-24 treatments during both the warm and cold seasons (Fig. 4a,c). The lowest CO_2_ efflux values with respect to pika burrow density were recorded under the RG-24 treatment: ~150 and 100 pika burrows/ha during the warm and cold seasons, respectively (Fig. 4a,c). There was also a positive linear relationship between CO_2_ efflux and zokor mound density under the CG-24 treatment during both the warm and cold seasons (Fig. 4b,d). During the warm season, the lowest CO_2_ efflux values were associated with ~150 and 270 zokor mounds/ha under the RG-24 and RG-48 treatments, respectively (Fig. 4b). During the cold season, the lowest CO_2_ efflux values were found for ~700 and 900 zokor mounds/ha under RG-24 and RG-48 treatment, respectively (Fig. 4d).

CH_4_ efflux and pika burrow density showed significant quadratic convex relationships under all grazing treatments during both the warm and cold seasons (Fig. 4e,g). The lowest CH_4_ efflux values were found for ~ 80, 120, and 170 burrows/ha under the CG-24, RG-24, and RG-48 treatments, respectively, during the warm season (Fig. 4e), and ~about 90, 100, and 150 burrows/ha during the cold season (Fig. 4g). There was a positive linear relationship between CH_4_ efflux and zokor mound density under the CG-24 treatment during both the warm and cold seasons (Fig. 4f,h). During the warm season, the lowest CH_4_ efflux values were associated with ~280 and 200 zokor mounds/ha under the RG-24 and RG-48 treatments, respectively (Fig. 4f). During the cold season, the lowest CH_4_ efflux values were associated with ~320 and 680 zokor mounds/ha under the RG-24 and RG-48 treatments, respectively (Fig. 4h).

A more comprehensive analytical approach was used evaluate a model that examines the relationship between GHG efflux and all of the factors (Table S2). The best fit in the regression of GHG efflux was achieved when socking rate, pika burrow density and zokor mound density were used as explanatory variables in cold season in 2010 (Table S2).

### Relationships between rodent density, GHG emissions and vegetation, soil properties

**Figure 5:**
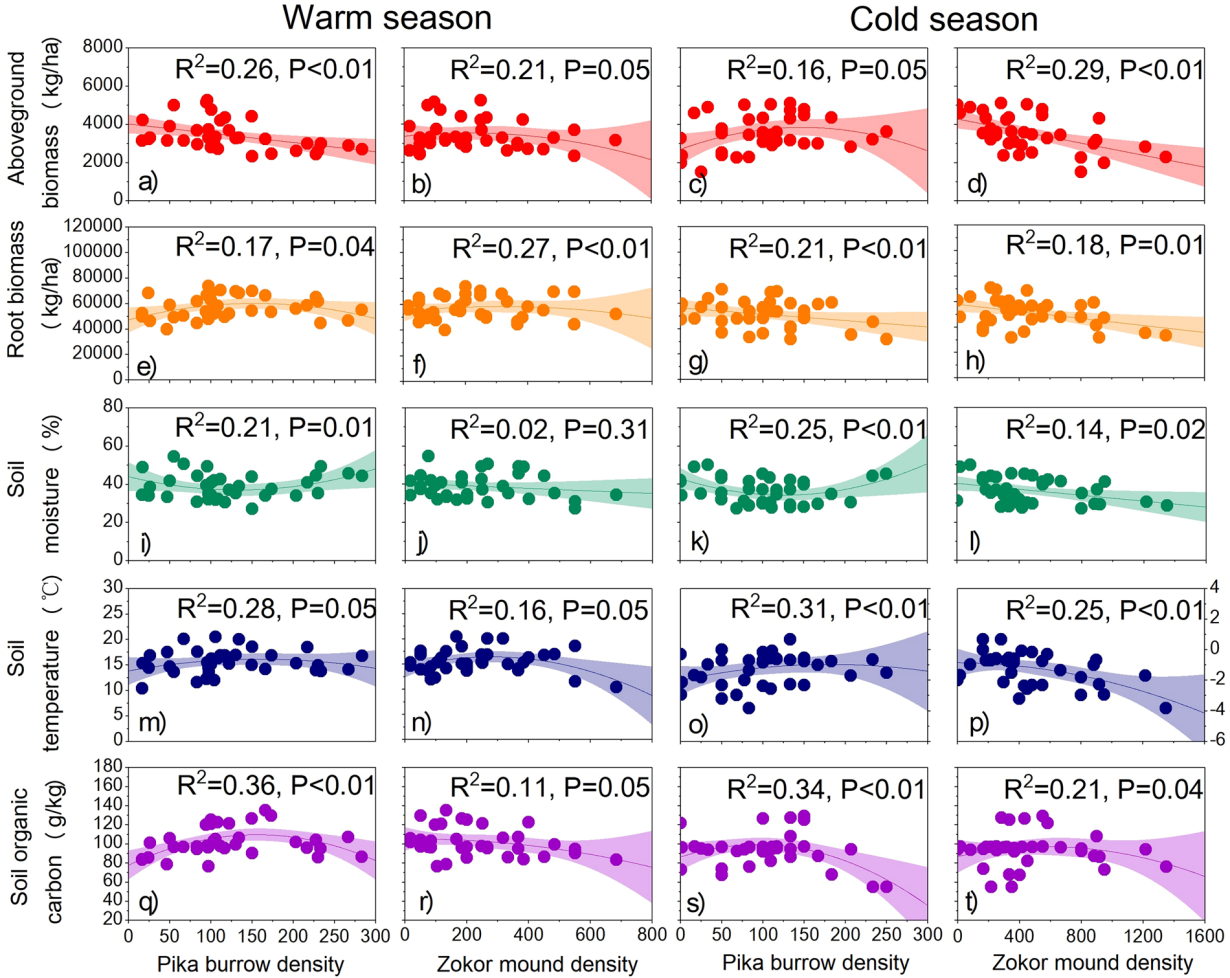
Relationships between aboveground biomass, root biomass, soil moisture, soil temperature, and SOC and rodent density (pika burrow and zokor mound density) during the warm and cold seasons. Lines denote the linear fit; shaded areas indicate confidence interval.

**Figure 6:**
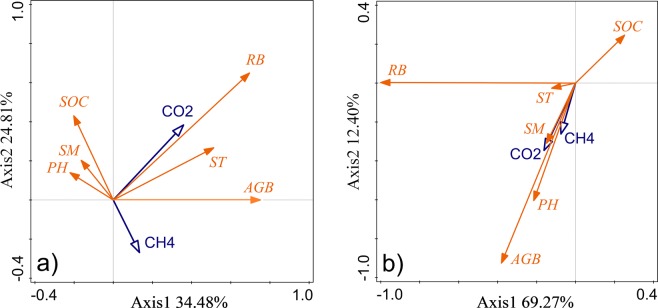
Biplot of the first two axes of the distance-based redundancy analysis for CO_2_ and CH_4_ efflux associated with various ecological characteristics. AGB: aboveground biomass; RB: root biomass; SM: soil moisture; ST: soil temperature; SOC: soil organic carbon; PH: plant height.

In both warm and cold seasons, aboveground biomass and root biomass showed significant negative linear or quadratic relationships with pika burrow density (Fig. 5a,c,e,g) and zokor mound density (Fig. 5b,d,f,h). Soil moisture had quadratic convex relationships with pika burrow density in both warm and cold seasons (Fig. 5i,k) and had significant negative linear relationships with zokor mound density in cold season (Fig. 5l). Soil temperature and soil organic carbon reached their maxima at a moderate rodent density (Fig. 5). The RDA analysis in this study indicated that aboveground biomass and soil moisture are good explanations for variations in GHG emissions. During warm and cold seasons, CO_2_ and CH_4_ effluxes were both positively correlated with aboveground biomass and soil temperature and negatively correlated with soil organic carbon (Fig. 6).

## Discussion

The impact of livestock grazing on GHG emissions has long been recognized^24–27^, but only a few studies have looked at the effect of different grazing management (e.g. rotational versus continuous grazing) on GHG emissions on the Tibetan grassland^28–30^. The results of this study indicate that CO_2_ efflux was significantly affected by grazing season and not grazing system (Fig. 2a,b), whereas CH_4_ efflux was significantly affected by grazing system and not grazing season (Fig. 2c,d). The net effects of grazing on CO_2_ efflux are determined by a balance of negative and positive effects from grazing on ecosystem respiration processes^31^. For example, the removal of herbage biomass from grazing reduces autotrophic respiration by plants but increases soil temperature, which in turn increases soil respiration^32^. In this study, there was no significant difference in residual herbage biomass or soil temperature between rotational and continuous grazing (Fig. 1c). This lends a plausible explanation for the lack of an effect of grazing system on CO_2_ efflux. The seasonal variations of CO_2_ efflux were driven by the soil temperature, which is known to be a primary factor controlling ecosystem respiration^33^. Soil moisture strongly controls CH_4_ dynamics, because CH_4_ production occurs under anaerobic conditions and thus requires saturated soil^34^. In this study, soil moisture under rotational grazing at 24 SM/ha was higher than that under rotational grazing at 48 SM/ha and continuous grazing in both warm and cold seasons (Fig. 1b). Saggar *et al*.^35^ also indicated that rotational grazing reduced CH_4_ flux due to trampling by livestock, indicating that soil compaction may decrease O_2_ diffusion into the soil and limit CH_4_ and O_2_ availability for oxidation. Our study lacks data on soil microbes (e.g. soil microbe activity, bacterial methane oxidation), which have substantial impacts on CO_2_ and CH_4_ efflux^36^.

The burrowing and feeding activities of plateau rodents exert huge effects on the plant community and on soil properties^37,38^. Although there are limited studies on the effects of rodent activity on GHG emissions^7,8,22,23^, it is known that rodent foraging and mound-building change plant species and significantly reduce the belowground plant biomass, soil organic matter pools, microbiological activity, and soil aggregation, which in turn can affect GHG emissions^6,39^. There are also no reports on the relationship between rodent population density and GHG emissions in areas using different grazing systems. The results of this study indicate that GHG emissions (CO_2_ and CH_4_ efflux) increased linearly with population density (pika burrows and zokor mounds) under seasonal continuous grazing in warm and cold seasons (Figs. 4, S1 and S2). Previous studies in alpine grassland with traditional continuous grazing showed that high soil respiration occurs in areas of high pika density because excreta deposited by pika in or near their burrows stimulates soil microbial activity^28,40,41^. Additionally, pika frequently haunted in or near active pika holes, which disturbs the soil structure and thus makes organic carbon available to microbial decomposition, leading to an increase in ecosystem CO_2_ emission^8,22^. In this study, GHG emissions and rodent population density showed a significant quadratic convex relationship under rotational grazing in warm and cold seasons except for CH_4_ efflux relative to pika density with the RG-48 grazing treatment during the warm season (Figs. 4, S1 and S2). Soil respiration and ecosystem respiration in alpine meadows decrease with increasing pika density because of the effect of rodent activity on soil carbon and nitrogen levels, which in turn regulate grassland biomass^23^. When zokor mound density is higher, improvements in soil gas permeability and aeration and a decrease in soil moisture facilitates diffusion of atmospheric CH_4_ and oxygen into the soil. Sufficient CH_4_ and oxygen substrates for methanotrophic bacteria in zokor mounds enhance atmospheric CH_4_ uptake^42^. The RDA analysis in this study indicated that aboveground biomass and soil moisture are good explanations for variations in GHG emissions. During warm and cold seasons, CO_2_ and CH_4_ effluxes were both positively correlated with aboveground biomass and soil temperature and negatively correlated with soil organic carbon (Fig. 6). Furthermore, aboveground biomass and root biomass showed significant negative linear or quadratic relationships with rodent density (Fig. 5). Soil moisture had a quadratic convex relationship with rodent density (Fig. 6). Soil temperature and soil organic carbon reached their maxima at a moderate rodent density (Fig. 5).

**Figure 7:**
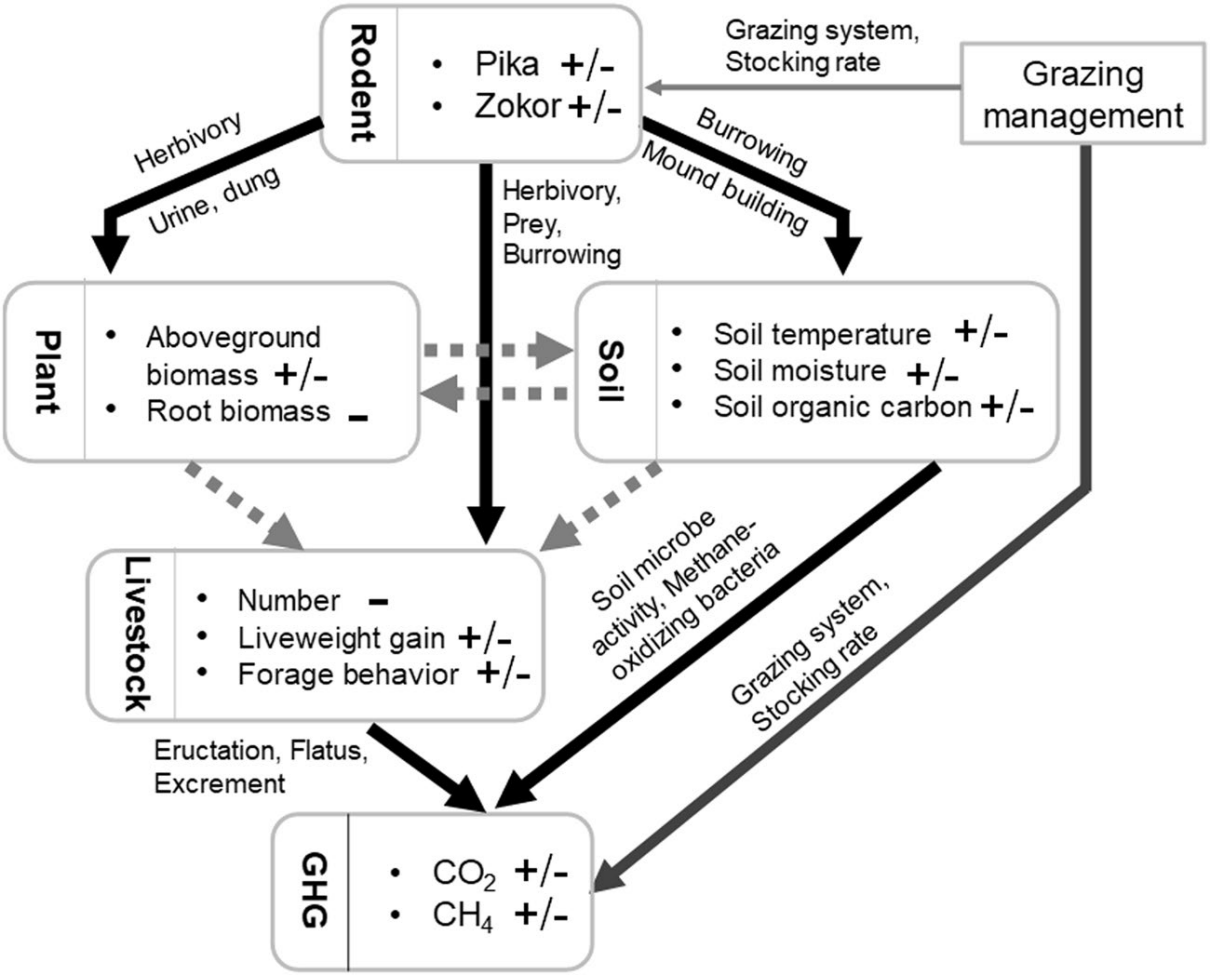
A conceptual framework for the effect of rodents on GHG emissions through plant, soil, and livestock processes. + and − signs represent an increase and decrease, respectively; +/− indicates an increase or a decrease.

Figure 7 presents a conceptual framework of possible pathways and mechanisms by which rodents affect GHG emission in the alpine meadow ecosystem. There are key features of this model. (i) By digging holes, generating mounds, and clipping vegetation, rodents affect the aboveground biomass, which affects soil temperature, moisture, and organic carbon and also affects livestock foraging behavior through competition for resources. (ii) Soil microbe activity (which is influenced by plant and soil properties) and livestock eructation, flatulence, and excrement all regulate GHG emissions. (iii) Both rodent activity and GHG emissions are controlled by grazing management.

Native rodent species are often perceived as pests when their behavior results in degradation of grassland and other consequences that adversely impact the economic wellbeing of local herders^43,44^, especially when rodent population densities are high^45,46^. However, rodents also play key roles in grassland ecosystems and help maintain grassland biodiversity^47^. Our study demonstrates that the lowest GHG emissions were observed at optimal pika and zokor population densities and that the relationship between GHG emissions and rodent population density was regulated by grazing practices (Fig. 4). This supports the notion that appropriate rodent populations are beneficial for soil carbon sequestration in alpine meadows. Further analysis of this phenomenon should include data on the quantitative interactive effects of livestock and rodents on GHG emission. Based on our findings, we suggest that populations of both domestic livestock and native burrowing mammals can be sustained on grasslands if numbers and grazing practices are properly managed and that they may interact synergistically to enhance the multifunctionality of these important grassland ecosystems.

## Conclusions

Grazing season significantly affected CO_2_ efflux, whereas grazing system significantly affected CH_4_ efflux. CO_2_ and CH_4_ efflux and rodent population density were showed significant relationships and these relationships were regulated by grazing management. Under rotational grazing at light stocking rate (24 SM/ha), appropriate populations of rodents (pika and zokor) were beneficial for decreasing GHG emissions. This results also used to help drive a best-practices model for grazing practices of local herders. A conceptual framework of possible pathways and mechanisms by which rodents affect GHG emission was proposed.

## Methods

### Experimental site

**Figure 8:**
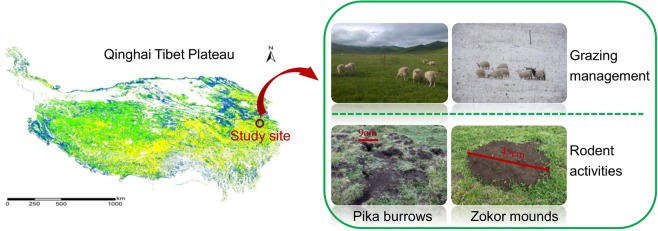
Location of study site on the Qinghai Tibet Plateau. The QTP is bordered by Sichuan and Yunnan Provinces in the southeast and by Gansu and Xinjiang Provinces in the north and northwest. The photographs in Fig. 1 were taken by Yingxin Wang.

^48^. The annual cloud-free sunshine is about 2,580 h. Species forming a major component of the vegetation in the study area include: *Rhizomatous Kobresia* spp. (*Cyperaceae*); *Festuca ovina*, *Poa poophagorum*, *Roegneria nutans*, and *Agrostis* spp. (*Poaceae*); *Saussurea* spp. (*Asteraceae*); and *Anemone rivularis (Ranunculaceae*). The average aboveground biomass is 700–1,000 kg DM ha^–1^. Typically, there are 25–35 vascular plant species and 800–1,000 individual plants per square meter^49^.

This study was conducted on a 20-ha botanically diverse, fenced alpine meadow located on the eastern QTP at the field station of Lanzhou University the Maqu County Azi Livestock Breeding Base (latitude 35°58′N, 101°53′E; elevation, 3,750 m) in the Gannan prefecture, Gansu Province, China (Fig. 8). This area has a frost-free period of 90–100 days with an annual mean air temperature of 1.2 °C, a monthly mean maximum of 11.7 °C in July, and a monthly mean minimum of −10 °C in January. The mean annual precipitation of approximately 620 mm is distributed unevenly among seasons, falling primarily as rain during the short, cool summer

### Grazing trials

Tibetan sheep grazing trials were set up at the beginning of the study (late April, 2010). Three grazing treatments were analyzed. (i) Warm-season rotational grazing was performed at the field station at 24 and 48 sheep months (SM)/ha (eight sheep grazed from July to September in 1.0-ha and 0.5-ha paddocks, respectively). At each stocking rate treatment, there were six replicates (ii) Cold-season rotational grazing was performed at the field station at 24 and 48 SM/ha (eight sheep grazed from October to December in 1.0-ha and 0.5-ha paddocks, respectively). At each stocking rate treatment, there were six replicates. (iii) Seasonal continuous grazing was performed at the field station at 24 SM/ha (eight sheep grazed from July to December in each of three 2.0-ha replicate paddocks). Within each replicate, the warm-season stocked paddocks were subdivided into three sub-paddocks and the cold-season stocked paddocks were subdivided into two sub-paddocks. Sheep were moved between the sub-paddocks every 10 and 15 days in the warm season rotational grazing and cold-season rotational grazing seasons, respectively. There were no subdivided paddocks in the replicates of seasonal continuous grazing treatment. For full details of sheep management, see Sun *et al*.^50^ and Du *et al*.^51^.

### Estimation of pika and zokor population density

Pika and zokor activity was observed and recorded three times during each trial year (2010 and 2011): early July (beginning of the warm season), early October (end of the warm season and beginning of the cold season), and end of December (middle of the cold season). In each paddock, three 25-m × 25-m observation zones were pegged out (July, 2010) as representative samples of replicate paddock. It is difficult to quantify the actual rodent’s population densities in the field. Generally, active entrance for plateau pika and mound for plateau zokor are usually used to indicate the relative population densities. The higher active burrow entrances and mounds per plot meant the higher disturbance intensity of plateau pika and plateau zokor. We estimated by counting active pika burrow entrances and zokor mounds as follows: pika: burrow entrances were plugged with sheets of newspaper and three days later, the number of plugs cleared by pika were counted; zokor: all mounds, both beside and covering entrances, and of varying age, were counted.

### Measurement of GHG emissions

The GHG emissions in the field were measured by a static closed chamber method. The cube-shaped chambers (40 × 40 × 40 cm) were constructed from stainless steel. The chambers were sheathed in foam plastic for improved temperature stability and fitted with an internal fan to ensure complete gas mixing and with a port with a septum for gas sampling. For a gas seal at the soil surface, the bottom edge of each chamber was seated into a Y-shaped, water filled channel, with the lower arm of the “Y” penetrating the soil to approximately 7 cm depth. Gas sampling was performed with plastic syringes (20 ml capacity) fitted with three-way stopcocks and connected to multilayer foil/plastic sampling bags commercially available in China for storing gas samples collected for research purposes (Dalian Delin Gas Packaging Co. Ltd.).

Gas sampling was conducted in the warm season (Mid-August) and in the cold season (Late-November) in 2010 and 2011. In this study, the percentage that chambers were on vs. off mounds was based on the ratio of area of rodent mound to vegetated areas (2.04%). We calculated the value using the mound density and area of a mound in whole study site. In each of sub-paddock of rotational grazing, three locations (subsamples) were to measure the GHG gas. In each of paddocks of seasonal continuous grazing, three locations (samples) were to measure the GHG gas.

During gas collection, samples were withdrawn into the syringe and then, after switching the stopcock, injected immediately into the plastic gasbags. Four gas samples of approximately 250 ml were taken in each chamber at four-time intervals for each sampling event (0, 10, 20 and 30 min) from 9:00 am to 11:00 am at local time to represent daily mean flux. Temperatures inside the chamber and at soil or manure depths of 50 mm were also recorded on each sampling occasion. A CH_4_/CO_2_ analyzer with syringe injection (DLT-100, Model No. 908-0011-0001) was used for simultaneous CH_4_ and CO_2_ analysis. The fluxes were calculated according to the equation with modifications made for QTP conditions as follows:

$${\rm{F}}=\rho \cdot \frac{{\rm{V}}}{{\rm{A}}}\cdot \frac{{{\rm{P}}}_{{\rm{s}}}}{{{\rm{P}}}_{0}}\cdot \frac{{{\rm{T}}}_{0}}{{\rm{T}}}\cdot \frac{{{\rm{dC}}}_{{\rm{t}}}}{{{\rm{D}}}_{{\rm{t}}}}$$

where F is gas flux (mg m^−2^ h^−1^), ρ is gas density under standard conditions (1.977 and 0.717 kg m^−3^ for CO_2_ and CH_4_, respectively), V is chamber volume (m^3^), A is base area of the chamber (m^2^), Ps is atmospheric pressure (kPa) of the sampling sites, P_0_ is atmospheric pressure under standard conditions (101.325 kPa), T_0_ is temperature under standard conditions (273.15 K), T is temperature inside the chamber (K), and dCt/dt is the average rate of concentration change with time.

### Integration of GHG data to estimate CO_2_ equivalents (CO_2_-eq.)

Following the methodology of other published studies^7^, the combined impact of different sources and GHG is expressed in CO_2_ equivalents (CO_2_-eq.) based on GWP (global warming potential) factors compared to CO_2_ of 25 times for CH_4_ and 310 times for N_2_O for a time horizon of 100 years.

### Vegetation and soil measurements

Vegetation. After GHG emissions measurements were completed in each replicate plot, vegetation data were collected from the areas where the gas chambers were located in August (the warm season) and November (the cold season) of 2010 and 2011. A 0.5-m × 0.5-m quadrat was designated for assessing vegetation. All shoots within the quadrat were measured (height in centimeters) and collected, and all on-ground litter was removed and bagged together. Litter and shoots from each species were oven-dried separately at 65 °C for 48 h and then weighed. The total shoot weight represented the sum from individual species.

#### Soil properties

The surface layer (horizon A) temperature and soil moisture content of each sample were measured at a depth of 10 cm. The soil was sampled in the middle of each 0.5-m × 0.5-m quadrat using an auger with a 10-cm diameter. Soil was removed in 10-cm layers down to a depth of 40 cm, and each layer was placed in separate 2-mm mesh bags. After air-drying for 1 month in a glasshouse, the soil from each layer was divided into root and soil subsamples. Root subsamples were washed free of soil, oven-dried at 115 °C for 48 h, and weighed. Soil subsamples were air-dried in the laboratory at room temperature and sieved through a 0.2-mm mesh. SOC was measured by the Walkey and Black method^52^.

### Statistical analysis

All data were analyzed using SAS software version 9.3 (SAS Institute, Inc. Cary, NC, USA), with significance levels set at *P* < 0.05. A goodness-of-fit test (Shapiro-Wilk test) was used to test data distributions and confirm normality. ANOVA (Proc ANOVA) was used to assess differences in CO_2_ and CH_4_ efflux, pika burrow density, zokor mound density, root biomass, soil moisture, soil temperature, and SOC under different grazing management during the warm and cold seasons. General linear model (Proc GLM) was applied to determine the effects of year, grazing season, grazing system, stocking rate and their interactions on GHG emissions and rodent density. Simple linear regressions were computed to compare relationships between rodent density (pika burrow density and zokor mound density) and CO_2_ and CH_4_ efflux under each grazing management during the warm and cold seasons; to compare relationships between rodent density (pika burrow density and zokor mound density) and aboveground biomass, root biomass, soil moisture, soil temperature, soil organic carbon during the warm and cold seasons. Nonlinear regressions were computed to compare relationships between rodent density (pika burrow density, zokor mound density and their interactions) and CO_2_ and CH_4_ efflux under each grazing management during the warm and cold seasons. Distance-based redundancy analysis (db-RDA) was used to further assess the effects of ecological characteristics (aboveground biomass, root biomass, soil moisture, soil temperature, soil organic carbon, plant height) on CO_2_ and CH_4_ efflux during the warm and cold seasons. All figures were constructed using Sigma Plot 12.5 and Origin 9.1.

## Supplementary information

Supplementary information

## References

[1] Davidson AD, Lightfoot DC (2006). Keystone rodent interactions: prairie dogs and kangaroo rats structure the biotic composition of a decertified grassland. Ecography..

[2] Campos CM (2017). Role of small rodents in the seed dispersal process: *Microcavia australis* consuming *prosopis flexuosa* fruits. Austral Ecol..

[3] Pech RP, Arthur AD, Zhang YM, Lin H (2007). Population dynamics and responses to management of plateau pikas *Ochotona curzoniae*. J Appl Ecol..

[4] Prugh LR, Brashares JS (2012). Partitioning the effects of an ecosystem engineer: kangaroo rats control community structure via multiple pathways. J Anim Ecol..

[5] Vandegehuchte ML, Schütz M, de Schaetzen F, Risch AC (2017). Mammal-induced trophic cascades in invertebrate food webs are modulated by grazing intensity in subalpine grassland. J Anim Ecol..

[6] Zhang Y, Liu J (2003). Effects of plateau zokors (*Myospalax fontanierii*) on plant community and soil in an alpine meadow. J Mammal..

[7] Liu Y (2013). Effects of plateau pika (*Ochotona curzoniae*) on net ecosystem carbon exchange of grassland in the three rivers headwaters region, Qinghai-Tibet, China. Plant Soil..

[8] Qin. Y, Chen J, Yi S (2015). Plateau pikas burrowing activity accelerates ecosystem carbon emission from alpine grassland on the Qinghai-Tibetan Plateau. Ecol Eng..

[9] Wang TC (2008). Four-year dynamic of vegetation on mounds created by zokors (*Myospalax baileyi*) in a subalpine meadow of the Qinghai-Tibet Plateau. J Arid Environ..

[10] Yu C (2017). Soil disturbance and disturbance intensity: response of soil nutrient concentrations of alpine meadow to plateau pika bioturbation in the Qinghai-Tibetan Plateau, China. Geoderma..

[11] Pi, N. L. Study on the feeding habits of the plateau pika. In: Xia, W. P. ed. *Contributions to rodents control and rodents’ biology*. Beijing. Science Press, 1, 91–102 (1973)

[12] Fan, N., Zhou, W., Wei, W., Wang, Q. & Jiang, Y. Rodent pest management in the Qinghai–Tibet alpine meadow ecosystem. In: Singleton, G., Hinds, L., Leirs, H. & Zhang, Z. (eds), *Ecologically-Based Rodent Management*. Australian Centre for International Agricultural Research, Canberra, Australia, pp. 285–304 (1999).

[13] Smith AT, Foggin JM (1999). The plateau pika (*Ochotona curzoniae*) is a keystone species for biodiversity on the Tibetan plateau. Anim Conserv..

[14] Zhang YM, Zhang ZB, Liu JK (2010). Burrowing rodents as ecosystem engineers: the ecology and management of plateau zokors *Myospalax fontanierii* in alpine meadow ecosystems on the Tibetan Plateau. Mammal Rev..

[15] Ni J (2002). Carbon storage in grasslands of China. J Arid Environ..

[16] Tang XL (2018). Carbon pools in China’s terrestrial ecosystems: New estimates based on an intensive field survey. Proc Natl Acad Sci USA.

[17] Ganjurjav H (2016). Differential response of alpine steppe and alpine meadow to climate warming in the central Qinghai–Tibetan Plateau. Agr Forest Meteorol..

[18] Zhang Z (2018). The response of lake area and vegetation cover variations to climate change over the Qinghai-Tibetan Plateau during the past 30 years. Sci Total Environ..

[19] Agapie A, Höns R (2013). Carbon dioxide fluxes dominate the greenhouse gas exchanges of a seasonal wetland in the wet–dry tropics of northern Australia. Agr Forest Meteorol..

[20] Tian YL (2017). A study of the gas environment in foraging tunnels of plateau zokor (*Myospalax baileyi*) in the eastern Qilian mountain region. Acta Theriologica Sinica..

[21] Chen SP (2018). Plant diversity enhances productivity and soil carbon storage. Proc Natl Acad Sci USA.

[22] Zhang W (2014). The increasing distribution area of zokor mounds weaken greenhouse gas uptakes by alpine meadows in the Qinghai-Tibetan Plateau. Soil Biol Biochem..

[23] Peng F, Quangang Y, Xue X, Guo J, Wang T (2015). Effects of rodent-induced land degradation on ecosystem carbon fluxes in alpine meadow in the Qinghai-Tibet Plateau, China. Solid Earth..

[24] Owensby CE, Auen HLM (2006). Fluxes of CO_2_ from grazed and ungrazed tallgrass prairie. Rangeland Ecol Manag..

[25] Thomas AD (2012). Impact of grazing intensity on seasonal variations in soil organic carbon and soil CO_2_ efflux in two semiarid grasslands in southern Botswana. Philos T R Soc B..

[26] Parsons AJ, Thornley JHM, Newton PCD, Rasmussen S, Rowarth JS (2013). Soil carbon dynamics: the effects of nitrogen input, intake demand and off-take by animals. Sci Total Environ..

[27] Louro A, Cárdenas LM, García MI, Báez D (2016). Greenhouse gas fluxes from a grazed grassland soil after slurry injections and mineral fertilizer applications under the Atlantic climatic conditions of NW Spain. Sci Total Environ..

[28] Cao G (2004). Grazing intensity alters soil respiration in an alpine meadow on the Tibetan Plateau. Soil Biol Biochem..

[29] Luo Y, Wan S, Hui D, Wallace LL (2001). Acclimatization of soil respiration to warming in a tall grass prairie. Nature..

[30] Liu Y, Yan CY, Matthew C, Wood B, Hou FJ (2017). Key sources and seasonal dynamics of greenhouse gas fluxes from yak grazing systems on the Qinghai- Tibetan Plateau. Sci Rep..

[31] Zhu XX (2015). Effects of warming, grazing/cutting and nitrogen fertilization on greenhouse gas fluxes during growing seasons in an alpine meadow on the Tibetan Plateau. Agr Forest Meteorol..

[32] Luo CY (2015). Impacts of seasonal grazing on net ecosystem carbon exchange in alpine meadow on the Tibetan Plateau. Plant and Soil..

[33] Hu Q (2008). Growing season ecosystem respirations and associated component fluxes in two alpine meadows on the Tibetan Plateau. J Integr Plant Biol..

[34] Lin X (2009). Fluxes of CO_2_, CH_4_, and N_2_O in an alpine meadow affected by yak excreta on the Qinghai-Tibetan plateau during summer grazing periods. Soil Biol Biochem..

[35] Saggar S, Hedley CB, Giltrap DL, Lambie SM (2007). Measured and modelled estimates of nitrous oxide emission and methane consumption from a sheep-grazed pasture. Agr Ecosyst Environ..

[36] Ogle K (2018). Hyperactive soil microbes might weaken the terrestrial carbon sink. Nature.

[37] Li XG (2009). Dynamics of soil properties and organic carbon pool in topsoil of zokor-made mounds at an alpine site of the Qinghai-Tibetan Plateau. Biol Fert Soils..

[38] Yong Z (2016). Responses of alpine vegetation and soils to the disturbance of plateau pika *(Ochotona curzoniae*) at burrow level on the Qinghai–Tibetan Plateau of China. Ecol Eng..

[39] Zhang WG, Hang XL, Yang L, Ma HY (2009). Patterns of change amongst plant functional groups along a successional status of zokor mounds in the Qinghai-Tibetan Plateau. New Zeal J Agr Res..

[40] Gurney CM, Prugh LR, Brashares JS (2015). Restoration of native plants is reduced by rodent-caused soil disturbance and seed removal. Rangeland Ecol Manag..

[41] Moorhead LC, Souza L, Habeck CW, Lindroth RL, Classen AT (2017). Small mammal activity alters plant community composition and microbial activity in an old-field ecosystem. Ecosphere..

[42] Tian H (2016). The terrestrial biosphere as a net source of greenhouse gases to the atmosphere. Nature..

[43] Delibes-Mateos M, Smith AT, Slobodchikoff CN, Swenson JE (2011). The paradox of keystone species persecuted as pests: a call for the conservation of abundant small mammals in their native range. Biol Conserv..

[44] Erb LP, Ray C, Guralnick R (2016). Determinants of pika population density vs. occupancy in the southern Rocky Mountains. Ecol Appl..

[45] Harris RB, Wang W, Badinqiuying, Smith AT, Bedunah DJ (2015). Herbivory and competition of Tibetan steppe vegetation in winter pasture: effects of livestock exclosure and plateau pika reduction. PloS One..

[46] Li XL (2013). Grassland degradation on the Qinghai Tibet Plateau: implications for rehabilitation. Land Degrad Dev..

[47] Davidson AD, Brown DJH (2012). Ecological roles and conservation challenges osocial, burrowing, herbivorous mammals in the world’s grasslands. Front Eco Environ..

[48] Wang YX, Hodgkinson KC, Hou FJ, Wang ZF, Chang SH (2018). An evaluation of government-recommended stocking systems for sustaining pastoral businesses and ecosystems of the alpine meadows of the Qinghai Tibetan Plateau. Ecol Evol..

[49] Wang YX, Sun Y, Wang ZF, Chang SH, Hou FJ (2018). Grazing management options for restoration of alpine grasslands on the Qinghai Tibet Plateau. Ecosphere..

[50] Sun Y, Angerer JP, Hou FJ (2015). Effects of grazing systems on herbage mass and liveweight gain of Tibetan sheep in eastern Qinghai-Tibetan Plateau, China. Rangeland J..

[51] Du WC, Yan T, Chang SH, Wang ZF, Hou FJ (2017). Seasonal hogget grazing as a potential alternative grazing system for the Qinghai-Tibetan Plateau: weight gain and animal behaviour under continuous or rotational grazing at high or low stocking rates. Rangeland J..

[52] Nelson, D.W. & Sommers, L. Total carbon, organic carbon, and organic matter. Part 2. Chemical and microbiological properties(methodsofsoilan2). In Page A. L. (Ed.), *Methods of soil analysis* (pp. 539–579). Madison, WI: American Society of Agronomy. (1982).

