# Supplementary material for: Tibetan sheep grazing modifies rodent density and their interactions effect on GHG emissions of alpine meadow
Source: Sci Rep. 2019 Nov 19;9:17066. doi: 10.1038/s41598-019-53480-z (PMC6863865; doi:10.1038/s41598-019-53480-z)
Supplement: Supplementary file 1 — Supplementary information [file 41598_2019_53480_MOESM1_ESM.pdf]

# Tibetan sheep grazing modifies rodent density and their interactions effect on GHG emissions of alpine meadow

Yingxin Wang[1], Hang Yuan[1], Xinglu Zhang[2], Yi Sun[1], Shenghua Chang[1], Guang Li[2], Fujiang Hou[1]*

[1]State Key Laboratory of Grassland Agro-ecosystems, Key Laboratory of Grassland Livestock Industry Innovation, Ministry of Agriculture and Rural Affairs; College of Pastoral Agriculture Science and Technology, Lanzhou University, Lanzhou, 730020, China

[2]College of Forestry, Gansu Agricultural University, Gansu Provincial Key Laboratory of Arid land Crop Science, Lanzhou, 730070, China.

*Correspondence and requests for materials should be addressed to F.H. (email: cyhoufj@lzu.edu.cn)

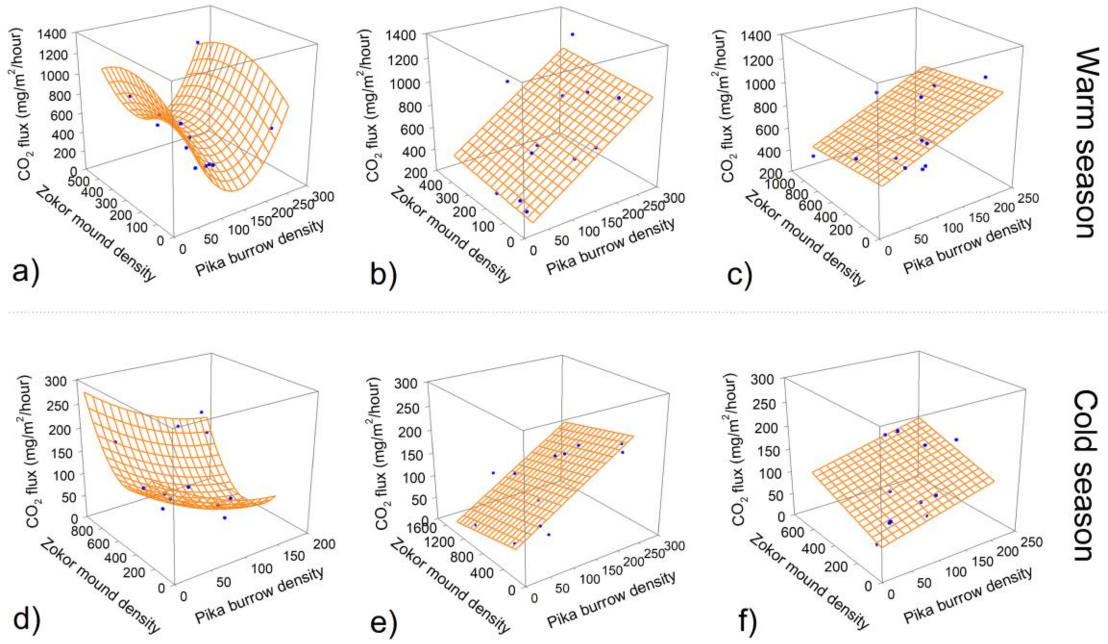


**Fig. S1 Relationships between $CO_2$ flux and rodent density (interaction of pika burrow and zokor mound density) under different grazing management during the warm and cold seasons.** Alpine meadows were grazed as in Fig. 2. Lines denote the nonlinear fit. (**a**, Warm-season rotational grazing at 24 SM/ha: $Y=0.0559X_1^2-0.0030X_2^2-15.75X_1+2.10X_2+1272.06$, $R^2=0.89$, $P=0.0003$; **b**, Warm-season rotational grazing at 48 SM/ha: $Y=2.77X_1+0.21X_2+201.57$, $R^2=0.61$, $P=0.0057$; **c**, Continuous grazing at 24 SM/ha in warm season: $Y=2.10X_1-0.08X_2+541.88$, $R^2=0.40$, $P=0.0483$; **d**, Cold-season rotational grazing at 24 SM/ha: $Y=0.0027X_1^2-0.0007X_2^2-1.13X_1-0.68X_2+294.79$, $R^2=0.69$, $P=0.0135$; **e,** Cold-season rotational grazing at 48 SM/ha: $Y=0.62X_1-0.03X_2+67.48$, $R^2=0.49$, $P=0.0467$; **f**, Continuous grazing at 24 SM/ha in cold season: $Y=0.29X_1+0.10X_2+72.56$, $R^2=0.70$, $P=0.0017$.

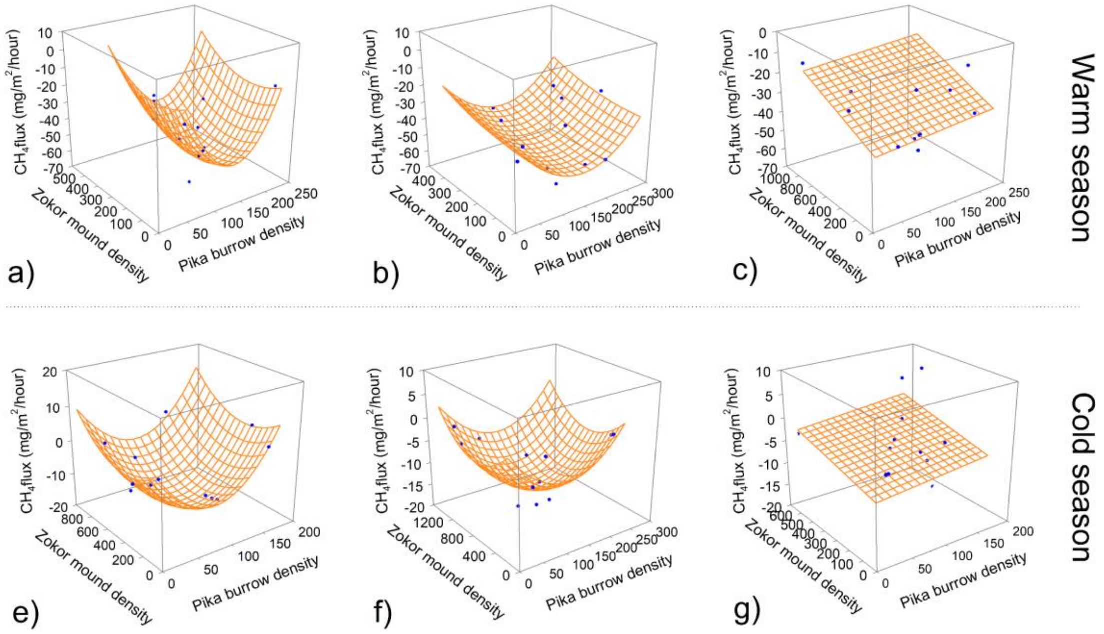


**Fig. S2 Relationships between $CH_4$ flux and rodent density (interaction of pika burrow and zokor mound density) under different grazing management during the warm and cold seasons.** Alpine meadows were grazed as in Fig. 2. Lines denote the nonlinear fit. (**a**, Warm-season rotational grazing at 24 SM/ha: $Y=-0.004X_1^2-0.0004X_2^2-3.37X_1-0.70X_2-32.44$, $R^2=0.44$, $P=0.0491$; **b**, Warm-season rotational grazing at 48 SM/ha: $Y=0.0013X_1^2+6.83E-5X_2^2-0.38X_1-0.043X_2-22.81$, $R^2=0.50$, $P=0.0470$; **c**, Continuous grazing at 24 SM/ha in warm season: $Y=0.0026X_1-0.023X_2-39.37$, $R^2=0.44$, $P=0.0444$; **d**, Cold-season rotational grazing at 24 SM/ha: $Y=-0.0016X_1^2-5.91E-5X_2^2-0.83X_1-0.18X_2-1.82$, $R^2=0.68$, $P=0.0137$; **e,** Cold-season rotational grazing at 48 SM/ha: $Y=-0.0006X_1^2-2.35E-5X_2^2-0.49X_1-0.10X_2-0.52$, $R^2=0.45$, $P=0.0350$; **f**, Continuous grazing at 24 SM/ha in cold season: $Y=0.0144X_1+0.0084X_2-5.78$, $R^2=0.22$, $P=0.0314$.

**Table S1** Prediction equation of GHG emissions ($CO_2$ flux, $CH_4$ flux and $CO_2$-eq) using stocking rate (SR), pika burrow density (Pika) and zokor mound density (Zokor) in warm and cold seasons of 2010 and 2011.

| Year | Grazing season | Equation | $R^2$ | P value |
|---|---|---|---|---|
| **2010** | **warm season** | $CO_2$ flux = 38.72SR + 2.48Pika + 4.65Zokor - 0.14SR*Pika - 0.21SR*Zokor - 0.02Pika*Zokor + 0.0008SR*Pika*Zokor - 131.50 | 0.44 | 0.1278 |
| | | $CH_4$ flux = -4.71SR - 0.33Pika - 0.38Zokor + 0.02SR*Pika + 0.015SR*Zokor + 0.0013Pika*Zokor - 4.8E-5SR*Pika*Zokor + 93.89 | 0.84 | 0.1522 |
| | | $CO_2$-eq= -79.04SR - 5.96Pika - 4.99Zokor + 0.25SR*Pika + 0.11SR*Zokor + 0.017Pika*Zokor - 0.0003SR*Pika*Zokor + 2215.86 | 0.89 | 0.0418 |
| **2011** | **warm season** | $CO_2$ flux = 21.23SR + 11.88Pika + 6.61Zokor - 0.22SR*Pika - 0.13SR*Zokor - 0.06Pika*Zokor + 0.0012SR*Pika*Zokor - 776.59 | 0.86 | 0.0441 |
| | | $CH_4$ flux = 4.01SR + 1.38Pika + 1.25Zokor - 0.03SR*Pika - 0.03SR*Zokor - 0.0098Pika*Zokor + 0.00024SR*Pika*Zokor - 219.05 | 0.39 | 0.1804 |
| | | $CO_2$-eq= 121.48SR + 46.46Pika + 38.08Zokor - 0.99SR*Pika - 0.90SR*Zokor - 0.31Pika*Zokor + 0.0072SR*Pika*Zokor - 6252.79 | 0.38 | 0.1867 |
| **2010** | **cold season** | $CO_2$ flux = 10.20SR + 4.79Pika + 0.80Zokor - 0.08SR*Pika - 0.006SR*Zokor - 0.009Pika*Zokor + 0.00018SR*Pika*Zokor - 506.17 | 0.98 | 0.0027 |
| | | $CH_4$ flux = 2.30SR + 0.69Pika + 0.15Zokor - 0.014SR*Pika - 0.004SR*Zokor - 0.0092Pika*Zokor + 2.24E-5SR*Pika*Zokor - 109.04 | 0.98 | 0.0045 |
| | | $CO_2$-eq= 67.778SR + 22.07Pika + 4.63Zokor - 0.44SR*Pika - 0.11SR*Zokor - 0.03Pika*Zokor + 0.0070SR*Pika*Zokor - 3232.58 | 0.98 | 0.0034 |
| **2011** | **cold season** | $CO_2$ flux = -13.11SR - 8.03Pika - 0.22Zokor + 0.20SR*Pika - 0.009SR*Zokor + 0.008Pika*Zokor - 0.0002SR*Pika*Zokor + 481.46 | 0.80 | 0.0457 |
| | | $CH_4$ flux = -0.41SR + 0.239Pika + 0.002Zokor - 0.004SR*Pika - 5.99E-5SR*Zokor - 0.0004Pika*Zokor + 8.6E-6SR*Pika*Zokor + 7.64 | 0.45 | 0.1161 |
| | | $CO_2$-eq= -23.41SR - 2.16Pika - 0.16Zokor + 0.08SR*Pika + 0.01SR*Zokor - 0.002Pika*Zokor + 1.97E-5SR*Pika*Zokor + 672.38 | 0.52 | 0.0806 |
